# The Involvement of Renin-Angiotensin System in Lipopolysaccharide-Induced Behavioral Changes, Neuroinflammation, and Disturbed Insulin Signaling

**DOI:** 10.3389/fphar.2019.00318

**Published:** 2019-04-02

**Authors:** Xiaoxue Gong, Hui Hu, Yi Qiao, Pengfei Xu, Mengqi Yang, Ruili Dang, Wenxiu Han, Yujin Guo, Dan Chen, Pei Jiang

**Affiliations:** ^1^Institute of Clinical Pharmacy and Pharmacology, Jining First People’s Hospital, Jining Medical University, Jining, China; ^2^Department of Cardiology, Jining First People’s Hospital, Jining Medical University, Jining, China; ^3^Department of Public Health, Jining Medical University, Jining, China

**Keywords:** lipopolysaccharide, depression, renin-angiotensin system, inflammation, insulin pathway

## Abstract

Brain insulin signaling is accounted for the development of a variety of neuropsychiatric disorders, such as anxiety and depression, whereas both inflammation and the activated renin-angiotensin system (RAS) are two major contributors to insulin resistance. Intriguingly, inflammation and RAS can activate each other, forming a positive feedback loop that would result in exacerbated unwanted tissue damage. To further examine the interrelationship among insulin signaling, neuroinflammation and RAS in the brain, the effect of repeated lipopolysaccharide (LPS) exposure and co-treatment with the angiotensin II (Ang II) receptor type 1 (AT1) blocker, candesartan (Cand), on anxiety and depression-like behaviors, RAS, neuroinflammation and insulin signaling was explored. Our results demonstrated that prolonged LPS challenge successfully induced the rats into anxiety and depression-like state, accompanied with significant neural apoptosis and neuroinflammation. LPS also activated RAS as evidenced by the enhanced angiotensin converting enzyme (ACE) expression, Ang II generation and AT1 expression. However, blocking the activated RAS with Cand co-treatment conferred neurobehavioral protective properties. The AT1 blocker markedly ameliorated the microglial activation, the enhanced gene expression of the proinflammatory cytokines and the overactivated NF-κB signaling. In addition, Cand also mitigated the LPS-induced disturbance of insulin signaling with the normalized phosphorylation of serine 307 and tyrosine 896 of insulin receptor substrate-1 (IRS-1). Collectively, the present study, for the first time, provided the direct evidence indicating that the inflammatory condition may interact with RAS to impede brain insulin pathway, resulting in neurobehavioral damage, and inhibiting RAS seems to be a promising strategy to block the cross-talk and cut off the vicious cycle between RAS and immune system.

## Introduction

Renin-angiotensin system (RAS) is originally acknowledged for its role in the regulation of blood pressure, but now it is generally accepted that brain has its intrinsic RAS with the major components, including angiotensin converting enzyme (ACE) and angiotensin II (Ang II) receptor type 1 (AT1), widely distributed in the central nervous system (Haspula and Clark, 2018; Uijl et al., 2018). The brain RAS actively participates in various neurological functions, including cognition, memory, emotion and stress response, and RAS over-activation has been identified in several neuropsychiatric disorders, including Alzheimer’s disease, epilepsy and depression (Yagi et al., 2013; Gebre et al., 2018). Intriguingly, these disorders are also frequently characterized with neurodegeneration, inflammation and brain insulin resistance. In this context, the interrelationship between RAS and these neuropathological progresses is attracting increasing attention, since Ang II is recognized as a pleiotropic factor locally metabolized in the brain (Saavedra, 2017).

It has been demonstrated that RAS stimulation mediates oxidative and inflammatory damage in the liver, the pancreas, the kidney and the brain (Capettini et al., 2012; Gaddam et al., 2014; Biancardi et al., 2017). By binding to AT1, Ang II also induces disturbed glucose metabolism and insulin resistance in the liver, adipocyte and pancreas (Favre et al., 2015). On the other hand, interference with RAS using either ACE inhibitors (ACEIs) or AT1 blockers confers protective effects against excessive inflammatory activation and improves insulin sensitivity (Luther and Brown, 2011; Favre et al., 2015). Despite the consensus that neuroinflammation and insulin resistance are two hallmarks of neuropathy, in both of which RAS plays an essential role, the evidence concerning intricate interrelationship among RAS, immune system and insulin signaling is limited and controversial, especially in the brain.

Therefore, in this study, we used repeated treatment of the endotoxin, lipopolysaccharide (LPS), to provoke sustained neuroinflammation, and explored the potential beneficial effects of AT1 receptor blocker, candesartan (Cand), treatment on LPS-induced abnormalities in behavioral changes, neuroimmune activation, and insulin signaling.

## Materials and Methods

### Animals

Male, Sprague-Dawley rats (200–230 g) were housed under standard conditions of temperature (23 ± 2°) and light (12:12 h light/dark cycle), with free access to standard rodent chow and water. Each rat was housed in a separate cage and recorded with body weight daily. All animal use procedures were carried out in accordance with the Regulations of Experimental Animal Administration issued by the State Committee of Science and Technology of the People’s Republic of China, with the approval of the Animal Ethics Committee of the Jining Medical University (NO. 20170037).

### Drug Treatment

**FIGURE 1:**
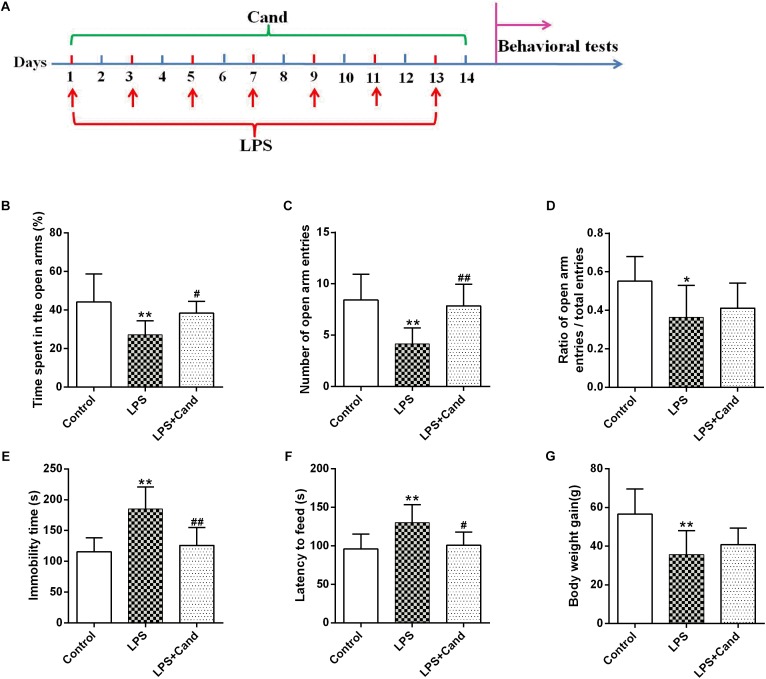
Timeline of experimental procedures **(A)** and effect of Candesartan (Cand) treatment on LPS-induced behavioral deficits in elevated plus maze (EPM) test **(B–D)**, forced swim test (FST) **(E)** and novelty-suppressed feeding test (NSFT) **(F)**, and body weight gain **(G)**. Data are means ± SD (*n* = 7–9). ^∗^*p* < 0.05, ^∗∗^*p* < 0.01 compared to Control group. ^#^*p* < 0.05, ^##^*p* < 0.01 compared to LPS group.

Rats were randomly divided into three groups: Control, LPS and LPS+Cand. Rats received LPS or normal saline via intraperitoneal injection at a dose of 500 μg/kg every 2 days for a total of 7 injections. The dose of LPS was chosen to effectively provoke depressive-like behaviors and neuroinflammation based on our previous research (Jiang et al., 2017; Yang et al., 2018). Animals in LPS+Cand group received daily Cand (1 mg/kg, dissolved in 1% DMSO) by oral gavage over 2 weeks covering the whole LPS treatment process, while the other two groups were treated with the vehicle (1% DMSO in saline). The timeline of the experimental protocol is depicted in Figure 1A. The dose of Cand was selected based the previous finding that at this dose, Cand effectively blocks central AT1 receptors (Benicky et al., 2011; de Souza Gomes et al., 2015). Body weight of these rats was monitored throughout the experiment, and the drug doses were adjusted accordingly.

### Behavioral Tests

#### Elevated Plus Maze (EPM) Test

EPM test was performed to evaluate the LPS-induced anxiety-like behavior in rats. In brief, the maze apparatus was a cross-shaped Plexiglas platform with two opposite open arms (OA, 50 × 10 cm) and two opposite closed arms (CA, 50 × 10 cm) with 40 cm walls, connected by a central platform (CP, 10 × 10 cm) and elevated 50 cm from the floor in a dimly lit room. The animals were placed at the center of the apparatus with its head facing toward an open arm. The total number of entries into the open and closed arms, and the time spent in each arm during the 5 min period were recorded with a video camera mounted vertically above the apparatus.

#### Forced Swim Test (FST)

The paradigm is based upon the evaluation of immobility, as a measure of behavioral despair in stressful and inescapable situations (Liao et al., 2018). In brief, each rat was placed in a Plexiglas cylinder (45 cm height, 25 cm diameter) containing approximately 35 cm of water (24 ± 1°) for 15 min. The rats were then dried and removed to their home cage. They were placed again in the cylinders 24 h later, and a 5 min swim test was conducted. Each test session was videotaped and the duration of immobility, which is defined as floating passively and only making slight movements to keep the head above water, was scored by an experienced observer blind to the experimental design.

#### Novelty-Suppressed Feeding Test (NSFT)

Before testing, rats were food deprived for 24 h in their home cages. The rats were placed in an open field (75 × 75 × 40 cm) with a small amount of food placed on a piece of white paper (10 × 10 cm) in the center. Animals were allowed to explore the open field for 8 min. The latency to feed, specifically the time it took for the rat to approach and take the first bite of the food, was recorded by a stopwatch.

### Histopathological Staining

Brains were collected and the hippocampus was rapidly dissected from representative animals in each group. The hippocampus was fixed in 10% phosphate-buffered paraformaldehyde and then embedded in paraffin, prepared for histopathological examination and immunohistochemical staining. The paraffin tissue blocks were prepared for sectioning at 5μm thickness by sledge microtome. The obtained tissue sections were stained by hematoxylin and eosin (H&E). The number of damaged cells characterized by contraction of the nucleus, cellular edema, vacuolization, and darkened nucleus, were counted. The presence of apoptosis was assessed by the terminal deoxynucleotidyl transferase-mediated FITC-dUTP nick end labeling (Tunel) method, which detects fragmentation of DNA in the nucleus during apoptotic cell death *in situ* (Zhou et al., 2018), following the manufacturer’s protocol (Keygen Biotech, Nanjing, China). The average ratio of total TUNEL-positive neurocyte number was calculated. This ratio represented the apoptotic index of the sample and was compared between groups.

### Immunofluorescent Staining

For the immunofluorescent histochemistry analysis, paraffin-embedded coronal sections of the hippocampus (6 μm thickness) were dewaxed in xylol, rehydrated, and rinsed in phosphate-buffered saline (PBS). Antigen retrieval was performed by boiling the sections on an electric stove in a citric acid buffer (0.01 mol/L, pH 6.0), followed by incubation with blocking 5% goat serum for 1 h at room temperature. The sections were then incubated with the primary antibody anti-IBA-1 (Abcam, 1:200). The sections were washed with PBS three times and stained with DAPI (Beyotime Biotechnology, China) to stain the cell nuclei. Immunofluorescent images were taken with an inverted fluorescence microscope (Olympus, Japan).

### Ang II Analysis

The hippocampus was homogenized and centrifuged at 9500 rpm for 20 min at 4°C. The supernatant was used for the measurement of Ang II by using a commercially available Enzyme-Linked Immunosorbent Assay (ELISA) kit (Cusabio, China).

### Real-Time PCR Analysis

Total RNA was extracted by using Trizol reagent (invitrogen, United States) following the manufacturer’s instructions. Quantitative PCR was performed on Bio-rad Cx96 Detection System (Bio-rad, United States) using SYBR green PCR kit (Applied Bio-systems, United States) and gene-specific primers (Table 1). Each cDNA was tested in triplicate. Thermo profile conditions were: 50°C for 2 min, 95°C for 10 min, 40 cycles of amplification at 95°C for 15 s and 60°C for 1 min. Relative quantitation for PCR product was normalized to β-actin as an internal standard.

**Table 1:** Primer sequences used for the qPCR analysis.

Gene	Sense primer (5′–3′)	Antisense primer (5′–3′)	Amplicon length (bp)
ACE	CAGAGGCCAACTG GCATTAT	CTGGAAGTTGCTCAC GTCAA	137
AT1	CACCCGATCACCGA TCAC	CAGCCATTTTATACCAATCT CTCA	110
IL-1β	AGGTCGTCATCATCC CACGAG	GCTGTGGCAGCTAC CTATGTCTTG	119
IL-6	CACAAGTCCGGA GAGGAGAC	ACAGTGCATCATCGCT GTTC	167
IL-10	GTTTTACCTGGTA GAAGTGATGCC	CCACTGCCTTGCTTTTA TTCTC	155
iNOS	AGTGGCAACAT CAGGTCGG	CGATGCACAACTGGG TGAAC	166
Cox2	GCATTCTTTGCC CAGCACTT	GTCTTTGACTGTGG GAGGAT	210
CD68	CCACAGGCAGCAC AGTGGACA	TCCACAGCAGAAGC TTTGGCCC	135
Arg-1	GGGAAAAGCCAAT GAACAGC	CCAAATGACGCATA GGTCAGG	148
CD206	AGTTGGGTTCT CCTGTAGCCCAA	ACTACTACCTGAGCCCA CACCTGCT	161
β-Actin	CATCCTGCGTCT GGACCTGG	TAATGTCACGCA CGATTTCC	116

### Western Blot Analysis

For western blotting analysis, total protein was prepared from the right hippocampus, and the protein concentrations were analyzed using Bradford method. Samples were loaded on precast 12% SDS-PAGE gels with approximately 50 μg protein in each lane. The following antibodies and concentrations were used over night at 4°C; p-IKKβ (Ser177) (Cell signaling, 1:1000), IKKβ (Cell signaling, 1:1000), IκB (Cell signaling, 1:1000), P65 (Cell signaling, 1:1000), IRβ (Abcam, 1:1000), p-IRS (Ser307) (Sigma-Aldrich, 1:1000), p-IRS (Tyr896) (Abcam, 1:1000), IRS (Abcam, 1:500), and β-actin (Proteintech, 66009-1-Ig; 1:4000). It was then probed with HRP-conjugated secondary antibody for 40 min. The film signals were digitally scanned and then quantified using Image J software. The signals were normalized to β-actin as an internal standard. Original images of Western blot are supplied in Supplementary Material.

### Data Analysis

Results from the experiment were expressed as means ± SD and analyzed using SPSS version 17.0 software. Normality of distribution was assessed by the Lilliefors test, and homogeneity of variance was tested with the Levene’s test. Differences between groups were determined by one-way analysis of variance (ANOVA) test, followed by LSD test for *post hoc* comparisons when equal variances were assumed. The prior level of significance was established at *p* < 0.05.

## Results

### Neuroprotective Effects of Cand Against LPS-Induced Behavioral Deficits and Neural Death

**FIGURE 2:**
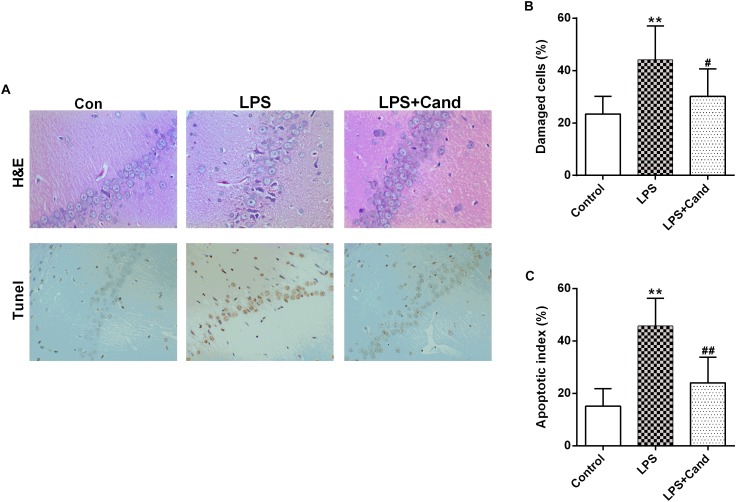
Effect of Candesartan (Cand) treatment on LPS-induced histopathological changes in H&E staining and apoptosis in Tunel test **(A)**. Damaged cells in H&E staining **(B)** and apoptotic cells in Tunel test were counted, respectively. Data are means ± SD (*n* = 7). ^∗∗^*p* < 0.01 compared to Control group. ^#^*p* < 0.05, ^##^*p* < 0.01 compared to LPS group.

As previously reported Guo et al. (2016) and Dang et al. (2017), repeated administration of LPS successfully induced an anxiogenic effect as evidenced by the decreased time spent in the open arms and reduced number and ratio of open arm entries (Figure 1B–D). LPS also induced the animals to a depression-like state with increased immobility time in FST (Figure 1E) and longer latency to feed in NSFT (Figure 1F). However, daily treatment of Cand partly restored the LPS-induced behavioral changes, increasing open arm spent time and open arm entries in the EPM test, and decreasing the immobility time and latency to feed in FST and NSFT, respectively, indicating both anxiolytic and antidepressive effects. While repeated LPS treatment suppressed the body weight growth, co-treatment with Cand didn’t significantly affect the body weight gain (Figure 1G). Additionally, we found Cand treatment ameliorated nuclear condensation and acidophilic degeneration in H&E histopathological examination. In consistence, by using Tunel method, the LPS-induced neural apoptosis was also mitigated by Cand treatment (Figure 2).

### Brain RAS Activation in LPS-Exposed Rats

**FIGURE 3:**
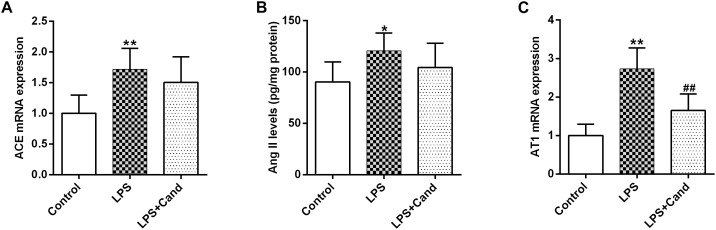
Effect of lipopolysaccharide (LPS) and Candesartan (Cand) treatment on ACE mRNA expression **(A)**, Ang II levels **(B),** and AT1 expression **(C)**. Data are means ± SD (*n* = 7). ^∗^*p* < 0.05, ^∗∗^*p* < 0.01 compared to Control group. ^##^*p* < 0.01 compared to LPS group.

As revealed in Figure 3, our results showed that sustained LPS exposure enhanced ACE expression, increased hippocampal status of Ang II and induced AT1 expression, suggesting that the continuous inflammatory process leads to brain RAS activation. Cand treatment had no effect on ACE and Ang II levels, but decreased AT1 expression compared with LPS-treated group.

### Anti-inflammatory Effect of Cand in LPS-Exposed Rats

**FIGURE 4:**
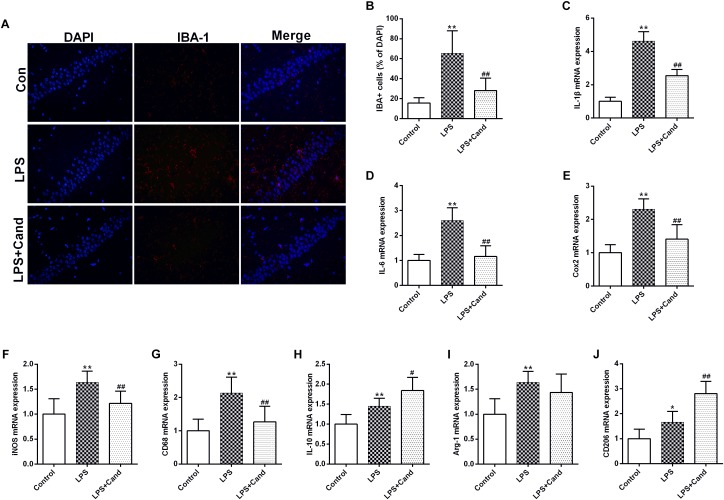
Anti-inflammatory effect candesartan (Cand) following repeated lipopolysaccharide (LPS) exposure. Microglial activation (Iba-1 immunofluorescence) **(A,B)** and mRNA expression of IL-1β **(C)**, IL-6 **(D)**, Cox2 **(E)**, iNOS **(F)**, CD68 **(G)**, IL-10 **(H)**, Arg-1 **(I)**, and CD206 **(J)**. Data are means ± SD (*n* = 7). ^∗^*p* < 0.05, ^∗∗^*p* < 0.01 compared to Control group. ^#^*p* < 0.05, ^##^*p* < 0.01 compared to LPS group.

In line with our previous findings (Jiang et al., 2017), LPS induced microglial activation as revealed by the IBA-1 immunofluorescent results, which was pronouncedly alleviated by Cand co-administration (Figure 4A). In accordance with the microglial activation, the hippocampal mRNA expression of pro-inflammatory cytokines, including IL-1β and IL-6, the anti-inflammatory cytokine, IL-10, and the inflammation mediating enzymes, including the nitric oxide (NO)-producing isoenzyme inducible NO synthase (iNOS) and cyclooxygenase 2 (COX-2) were synchronously increased, which were prevented by Cand administration except for IL-10 that Cand slightly but significantly further amplified the LPS-induced IL-10 expression (Figure 4B–F). Upon inflammatory stimulation, the microglia is prone to polarize into proinflammatory M1 phenotype and the proinflammatory cytokines are mediators or biomarkers of M1 cells (Zhang B. et al., 2018). Likewise, CD68, another biomarker of M1 phenotype was induced in LPS exposed group (Figure 4G), further supporting the notion that LPS stimuli would lead to M1 polarization. Interestingly, similar as IL-10, repeated LPS exposure also enhanced the expression of M2 markers, arginase-1 (Arg-1) and CD206, indicating a potential compensatory response (Figure 4H–J). Meanwhile, Cand treatment not only mitigated the LPS-induced M1 polarization, but also further shifted the microglia into M2 phenotype with increased expression of Arg-1 and CD206 compared with LPS group.

### The Effect of Cand on LPS-Induced NF-κB Signaling

**FIGURE 5:**
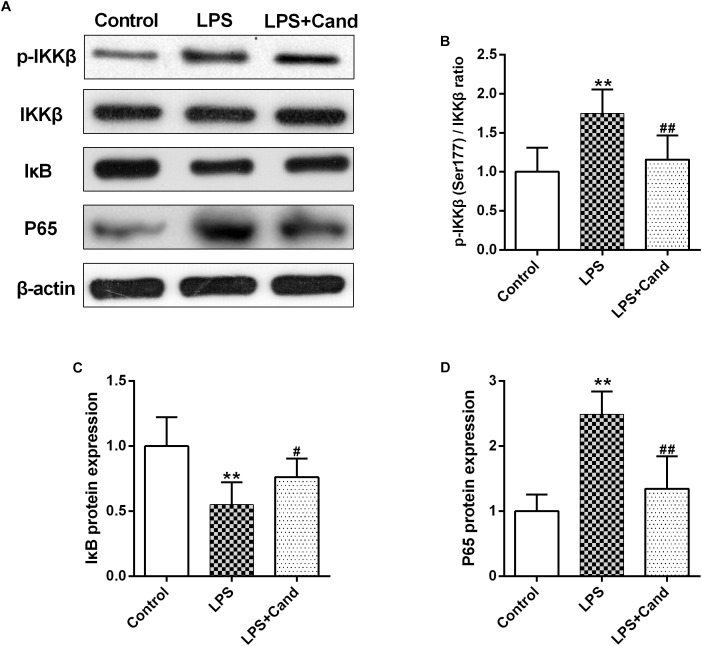
Effect of lipopolysaccharide (LPS) and Candesartan (Cand) treatment on NF-κB signaling. Representative blots **(A)** and statistical graphs of relative p-IKKβ (Ser177)/IKKβ ratio **(B)**, IκB protein expression **(C)** and P65 protein expression **(D)** (*n* = 7). Data are means ± SD. ^∗∗^*p* < 0.01 compared to Control group. ^#^*p* < 0.05, ^##^*p* < 0.01 compared to LPS group.

NF-κB signaling plays a fundamental role in response to inflammatory activator such as LPS (Lee et al., 2018). To further explore the mechanisms, we then analyzed the essential members of NF-κB family (Figure 5A). As expected, LPS stimuli activated IκBs kinase (IKK) with increased phosphorylation of IKK at serine 177 residue (Figure 5B), which resulted in IκB degradation (Figure 5C) and P65 activation (Figure 5D) compared with the control group. Cand treatment inhibited the LPS-induced IKK phosphorylation and P65 overactivation while preserved IκB stability. These data indicated that Cand may exert an anti-inflammatory effect by modulating the NF-κB signal pathway.

### The Effect of Cand and LPS on Hippocampal Insulin Pathway

**FIGURE 6:**
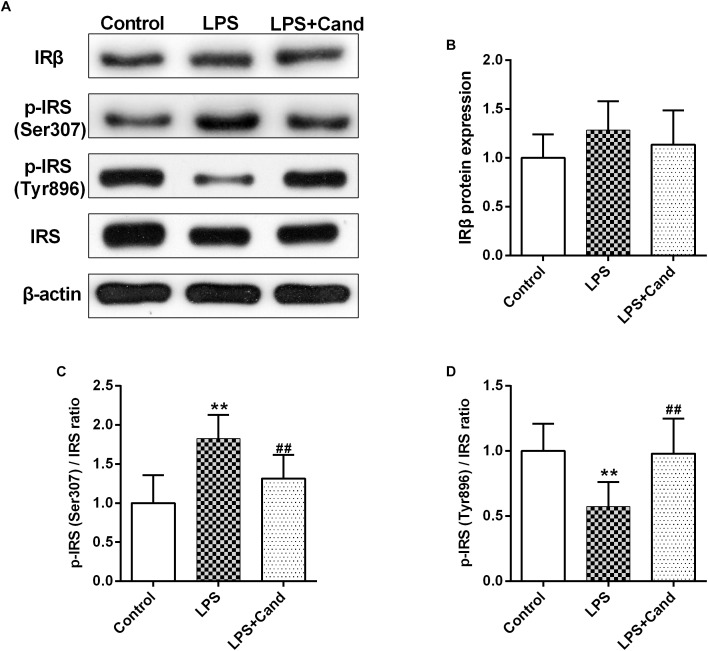
Effect of lipopolysaccharide (LPS) and Candesartan (Cand) treatment on insulin signaling. Representative blots **(A)** and statistical graphs of relative IRβ protein expression **(B)** and the ratios of p-IRS (Ser307)/IRS **(C)** and p-IRS (Tyr896)/IRS **(D)**. Data are means ± SD (*n* = 7). ^∗∗^*p* < 0.01 compared to Control group. ^##^*p* < 0.01 compared to LPS group.

Both inflammation and activated RAS are two major contributors to insulin resistance (Sarlus et al., 2013; Ramalingam et al., 2017). To examine their interactions in the brain, we evaluated the insulin signaling in the hippocampus following LPS and Cand treatment (Figure 6A). Although LPS and Cand both had no significant effects on IRβ expression (Figure 6B), repeated LPS treatment enhanced the inhibitory phosphorylation of IRS at serine 307 (Figure 6C) and suppressed IRS tyrosine 896 phosphorylation (Figure 6D) that is required for signal transduction (Akiyama et al., 2013), whereas blocking AT1 by Cand treatment reversed these effects and improved insulin signaling, partly normalizing p-IRS (Ser307) and p-IRS (Tyr896) status.

## Discussion

The progression of a series of neuropsychiatric disorders, such as depression, is associated with continuous subclinical inflammatory process. However, most of the studies examined the acute effect of LPS, and the evidence concerning the neuropathological changes following sustained inflammatory stimuli is limited. In line with our previous research, repeated administration of LPS induced anxiety and depression-like behaviors, as well as neuroinflammation, in rats, which resembles the clinical profile of depression that depressive patients are prone to under chronic subclinical inflammatory state (Dang et al., 2017). The neuroinflammatory process was reflected by the LPS-induced microglial activation, the enhanced expression of proinflammatory cytokines, IL-1β and IL-6, and the upregulated iNOS and Cox2 expression. Unexpectedly, the anti-inflammatory cytokine, IL-10, was also induced by prolonged exposure to LPS, which is in contrast with previous findings that acute LPS treatment inhibited IL-10 expression (Kavanagh et al., 2004), indicating a potential compensatory mechanism that may mediate endotoxin tolerance following sustained LPS treatment.

RAS functions as a hormone system that is capable of acting directly in many tissues in an autocrine and paracrine way. RAS is originally recognized as a blood pressure controller, and now is implicated in multifactorial brain function. All the components of the classic RAS have been identified in both neuronal and glial cells (Jackson et al., 2018). Microglia is considered as the resident macrophage in the brain, responsible for the neural immune responses under stressful conditions (Liu et al., 2019). The localized brain RAS activation is associated with neuroinflammation and neuropathy as demonstrated by the facts that central administration of Ang II, the major prohypertensive ligand of AT1 receptor, induces neuroinflammation and oxidative stress *in vivo* and *in vitro* (Bild et al., 2013; Bali and Jaggi, 2016; Abdul-Muneer et al., 2018), and AT1 receptor blockade ameliorates inflammation and improves brain function in animal models of epilepsy, brain ischemia and neurodegeneration (Saavedra et al., 2011; Tchekalarova et al., 2015; Valenzuela et al., 2016). Microglia, similar with macrophages, can develop into proinflammatory M1 phenotype and immunoregulatory M2 phenotype (Liu et al., 2019). In the context of immune stimuli, the localized RAS system in microglial cells would be activated, which plays an essential role in microglial polarization, promoting the transformation of microglia into M1 phenotype (Labandeira-Garcia et al., 2017). Consistent with this, our data showed the activated RAS in the brain with enhanced ACE and AT1 mRNA expression, and elevated Ang II status in the context of repeated LPS stimuli. In addition, our phenotypic analysis of microglia in the hippocampal CA1 region showed that the M1 markers iNOS and CD68 were increased. These findings are in line with acute LPS treatment (Benicky et al., 2011), indicating that the brain RAS can be ignited by both short and longtime neuroimmune activation, which may further worsen the inflammatory situation and trigger the positive feedback between RAS and immune system (Bhat et al., 2016), resulting in uncontrolled neuroinflammatory progression. Intriguingly, it should be noted that the M2 markers, IL-10, Arg-1, and CD206, were also synchronously increased following sustained LPS exposure, suggesting an adaptive compensatory response, which might be associated with the mechanism of LPS tolerance (Zhang Z. et al., 2018).

However, blocking AT1 receptor by Cand, as shown in our study and previous findings, contains anti-inflammatory properties (Bhat et al., 2016). While Cand can efficiently cross the blood brain barrier and effectively block brain AT1 receptor (Benicky et al., 2011), the treatment protocol didn’t significantly normalize the LPS-activated RAS except for the partly restored AT1 expression. Nevertheless, daily Cand administration during LPS challenge process attenuated the inflammatory response, suppressing microglial activation and the expression of inflammatory mediators. Additionally, Cand treatment shifted microglia from M1 to M2 phenotype with decreased expression of M1 markers (iNOS and CD68) and increased expression of M2 marker (CD206) compared with LPS group, further confirming the involvement of RAS system in the LPS-induced disturbance in microglial polarization. Aside from neuroimmune modulating actions, Cand treatment also prevented the brain from LPS-induced neural apoptosis and exerted anti-anxiety and anti-depression effects. The AT1 antagonism may cut off the deteriorating feedback loop between RAS and immune system, thereby resulting in neuroprotective activities and improving neurobehavioral functions. Although multiple lines of evidence suggest that blocking AT1 receptor contains anti-inflammatory, anti-oxidative and anti-apoptotic properties, it should be noted that Cand may interact with other targets independent of AT1 receptor. One previous interesting research showed that Cand effectively suppresses TNF-induced inflammatory process and reinstates redox homeostasis in renal tubular epithelial cells lacking AT1, and Cand may possess intrinsic antioxidant activity (Chen et al., 2008). To this end, further studies using AT1 knockdown animals or other AT1 inhibitors with more selectivity, such as azilsartan (Kajiya et al., 2011; Ojima et al., 2011), are warranted given that the pharmacological action of Cand seems to be complex.

Insulin resistance is a major consequence of inflammation, contributing to a variety of brain dysfunctions as glucose is the brain’s main energy source for neurotransmission, synaptic plasticity and neurogenesis (Clark et al., 2012; Kleinridders et al., 2014). Inflammatory stimuli may evoke insulin resistance via the activation of NF-κB signaling. Under normal condition, NF-κB exists in an inactive state in the cellular cytoplasm, where it is bound to the inhibitor of NF-κB (IκB). Once activated, the IκB kinase (IKK) phosphorylates IκB, which would lead to IκB degradation and P65 translocation to the nucleus, promoting the expression of proinflammatory mediators (Li et al., 2012). The activated IKK can also phosphorylate IRS-1 at serine residue, impeding tyrosine phosphorylation and insulin signal transduction (Copps and White, 2012). Although the cross-talk between insulin signaling and inflammation is well-studied in the periphery, the evidence concerning the relationship in the brain is limited. A recent study showed that single intraperitoneal LPS injection activates insulin signaling with enhanced IRS-1 tyrosine phosphorylation (Tyr1222) in the hypothalamus (Rorato et al., 2017). In contrast, Iloun et al. (2018) found that hippocampal insulin pathway is inhibited with increased IRS-1 (Ser307) expression 6 days after one dose of intracerebroventricular LPS injection. These discrepancies might be attributed to the treatment protocols and various brain areas used in the different study. The present study observed increased phosphorylated serine residue (Ser307) but decreased phosphorylated tyrosine residue (Tyr896), strongly indicating that continuous inflammatory state may impede insulin pathway, confirming the theory that inflammation interferes with insulin signaling to promote neurological and behavioral deficits, and IKK may mediate not only LPS-induced neuroinflammation, but also disturbance of insulin signaling in the brain.

Aside from inflammation, RAS may also hamper insulin signaling by directly inhibiting phosphatidylinositol 3-kinase (PI3K) cascade or indirectly through provoking inflammation and oxidative stress (Favre et al., 2015). Indeed, RAS is intrinsic to pancreatic islets and insulin-targeted tissues including adipose, skeletal muscle and liver, whereas both clinical and basic studies demonstrated that RAS blockade improves glucose homeostasis and prevents diabetes (Luther and Brown, 2011; Bangalore et al., 2016). In this context, we further explored whether angiotensin receptor blockers (ARBs) are also effective in brain insulin pathway. Our data showed that Cand alleviated LPS-induced IRS-1 phosphorylation on serine 307 and restored IRS-1 phosphorylation on tyrosine 896 in the hippocampus, implying that ARBs might be beneficial on brain insulin signaling as well. By blocking the AT1 receptor, Cand may restrain the reciprocal influence of RAS and inflammatory pathway on each other to limit the deleterious impacts of these two risk factors on insulin signaling.

In summary, the present study showed that LPS-induced anxiety and depression-like behaviors might be associated with brain RAS activation, neuroinflammation and disturbed brain insulin signaling, which were partly restored by Cand treatment, highlighting the involvement of RAS in inflammation-impeded insulin pathway in the brain and providing a potential drug target for the inflammation-associated neurological disorders such as depression. While the present research mainly focused on the neuroprotective mechanisms of Cand, it is important to note that we fails to evaluate the baseline effect of Cand, which is a major limitation of the study.

## Data Availability

The datasets generated for this study are available on request to the corresponding author.

## Author Contributions

XG, PJ, and YQ designed the study and wrote the protocol. XG, HH, PX, RD, MY, and WH performed the experiments and analyzed the data. XG, HH, and DC drafted the manuscript. PJ, MY, and YG revised the manuscript content. All authors read and approved the final manuscript.

## Conflict of Interest Statement

The authors declare that the research was conducted in the absence of any commercial or financial relationships that could be construed as a potential conflict of interest.
