# Supplementary material for: The Involvement of Renin-Angiotensin System in Lipopolysaccharide-Induced Behavioral Changes, Neuroinflammation, and Disturbed Insulin Signaling
Source: Front Pharmacol. 2019 Apr 2;10:318. doi: 10.3389/fphar.2019.00318 (PMC6454872; doi:10.3389/fphar.2019.00318)
Supplement: Supplementary file 1 [file Data_Sheet_1.zip › supple material/Figure caption.docx]

**Supplementary Figure captions**

**Supplementary Figure 1.** Full blots for p-IKKβ shown in Figure 5A.

**Supplementary Figure 2.** Full blots for IKKβ shown in Figure 5A.

**Supplementary Figure 3.** Full blots for IkB shown in Figure 5A.

**Supplementary Figure 4.** Full blots for P65 shown in Figure 5A.

**Supplementary Figure 5.** Full blots for β-actin shown in Figure 5A.

**Supplementary Figure 6.** Full blots for IRβ shown in Figure 6A.

**Supplementary Figure 7.** Full blots for p-IRS (Ser307) shown in Figure 6A.

**Supplementary Figure 8.** Full blots for p-IRS (Tyr896) shown in Figure 6A.

**Supplementary Figure 9.** Full blots for IRS shown in Figure 6A.

**Supplementary Figure 10.** Full blots for β-actin shown in Figure 6A.
