# Supplementary figures and images for: The Involvement of Renin-Angiotensin System in Lipopolysaccharide-Induced Behavioral Changes, Neuroinflammation, and Disturbed Insulin Signaling
Source: Front Pharmacol. 2019 Apr 2;10:318. doi: 10.3389/fphar.2019.00318 (PMC6454872; doi:10.3389/fphar.2019.00318)

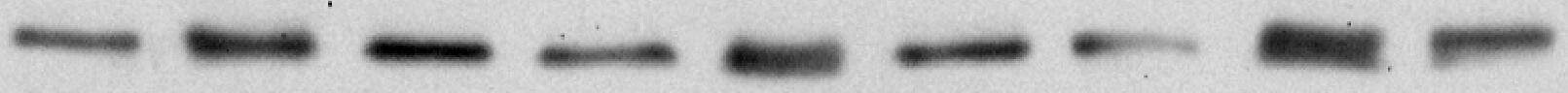

Supplement: Supplementary file 1 [file Data_Sheet_1.zip › supple material/Supplementary Figure 1, p-ikk.jpg]

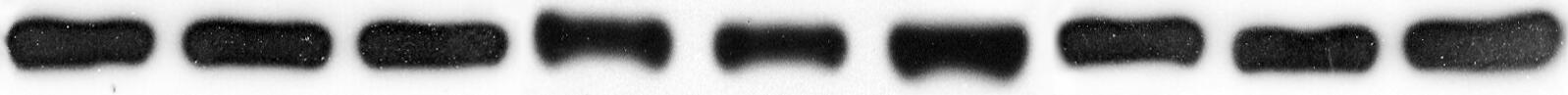

Supplement: Supplementary file 1 [file Data_Sheet_1.zip › supple material/Supplementary Figure 10, beta actin.jpg]

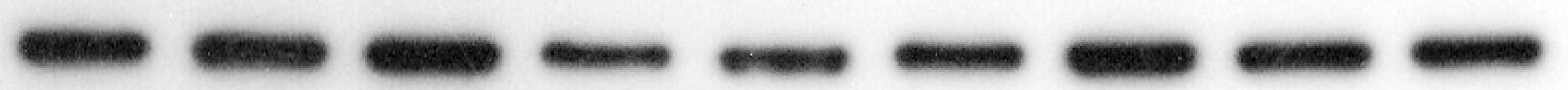

Supplement: Supplementary file 1 [file Data_Sheet_1.zip › supple material/Supplementary Figure 2, ikk.jpg]

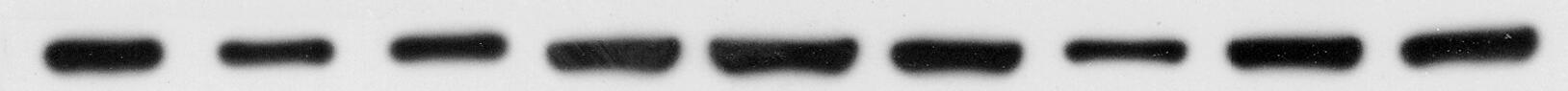

Supplement: Supplementary file 1 [file Data_Sheet_1.zip › supple material/Supplementary Figure 3, ikb.jpg]

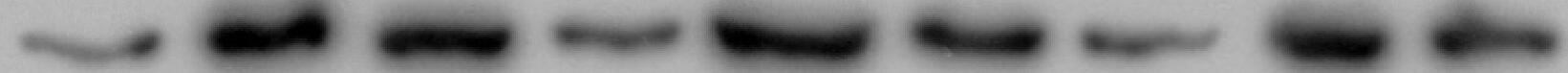

Supplement: Supplementary file 1 [file Data_Sheet_1.zip › supple material/Supplementary Figure 4, p65.jpg]

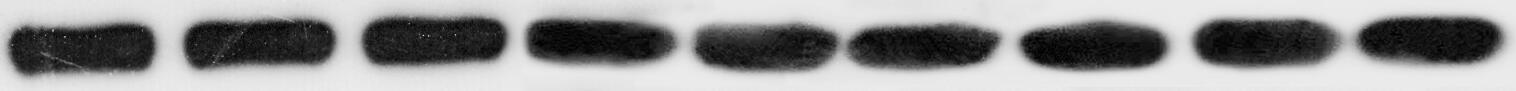

Supplement: Supplementary file 1 [file Data_Sheet_1.zip › supple material/Supplementary Figure 5, beta actin.jpg]

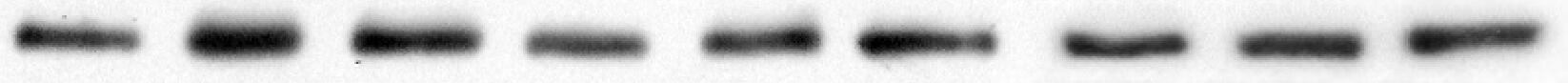

Supplement: Supplementary file 1 [file Data_Sheet_1.zip › supple material/Supplementary Figure 6, ir.jpg]

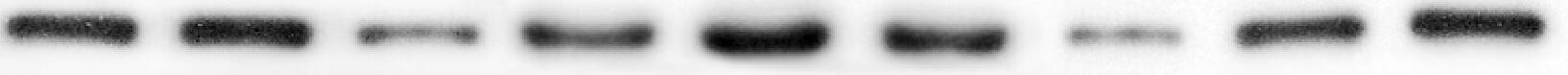

Supplement: Supplementary file 1 [file Data_Sheet_1.zip › supple material/Supplementary Figure 7, p-IRSSER.jpg]

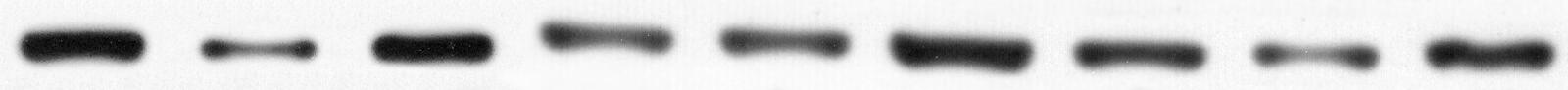

Supplement: Supplementary file 1 [file Data_Sheet_1.zip › supple material/Supplementary Figure 8, p-irstyr.jpg]

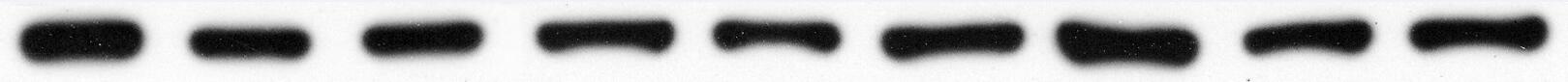

Supplement: Supplementary file 1 [file Data_Sheet_1.zip › supple material/Supplementary Figure 9, irs.jpg]
